# Green synthesis of zinc oxide nanoparticles using *Cocos nucifera* leaf extract: characterization, antimicrobial, antioxidant and photocatalytic activity

**DOI:** 10.1098/rsos.220858

**Published:** 2022-11-23

**Authors:** Farjana Rahman, Md Abdul Majed Patwary, Md. Abu Bakar Siddique, Muhammad Shahriar Bashar, Md. Aminul Haque, Beauty Akter, Rimi Rashid, Md. Anamul Haque, A. K. M. Royhan Uddin

**Affiliations:** ^1^ Department of Chemistry, Comilla University, Cumilla 3506, Bangladesh; ^2^ Department of Pharmacy, Comilla University, Cumilla 3506, Bangladesh; ^3^ Institute of National Analytical Research and Service (INARS), Bangladesh Council of Scientific and Industrial Research (BCSIR), Dhanmondi, Dhaka 1205, Bangladesh; ^4^ Institute of Fuel Research and Development (IFRD), Bangladesh Council of Scientific and Industrial Research (BCSIR), Dhanmondi, Dhaka 1205, Bangladesh; ^5^ Department of Chemistry, Jagannath University, Dhaka 1100, Bangladesh; ^6^ Materials Science Division, Atomic Energy Centre, Bangladesh Atomic Energy Commission, Dhaka 1000, Bangladesh

**Keywords:** ZnO NPs, *Cocos nucifera*, green approach, antimicrobial, antioxidant, photocatalyst

## Abstract

Zinc oxide nanoparticles (ZnO NPs) have been successfully prepared using *Cocos nucifera* leaf extract and their antimicrobial, antioxidant and photocatalytic activity investigated. The structural, compositional and morphological properties of the NPs were recorded and studied systematically to confirm the synthesis. The aqueous suspension of NPs showed an ultraviolet–visible (UV–Vis) absorption maxima of 370 nm, indicating primarily its formation. X-ray diffraction analysis identified the NPs with a hexagonal wurtzite structure and an average particle size of 16.6 nm. Fourier transform infrared analysis identified some biomolecules and functional groups in the leaf extract as responsible for the encapsulation and stabilization of ZnO NPs. Energy-dispersive X-ray analysis showed the desired elemental compositions in the material. A flower-shaped morphology of ZnO NPs was observed by scanning electron microscopy, with a grain size of around 15 nm. The optical properties of the NPs were studied by UV–Vis spectroscopy, and the band gap was calculated as 3.37 eV. The prepared ZnO NPs have demonstrated antimicrobial activity against *T. harzianum *and* S. aureus*, with a zone of inhibition of 14 and 10 mm, respectively. The photocatalytic behaviour of ZnO NPs showed absorbance degradation at around 640 nm and it discoloured methylene blue dye after 1 h, with a degradation maximum of 84.29%. Thus, the prepared ZnO NPs could potentially be used in antibiotic development and pharmaceutical industries, and as photocatalysts.

## 1. Introduction

Nanoscience and nanotechnology are the most emerging fields in recent times and are moving forward sharply, along with physics, chemistry, biology, molecular engineering and so on. Nanomaterials are in versatile use in pharmaceutical, cosmetic, textile, and even electrical and electronics industries. Nanomaterials are products processed through nanotechnologies that contain nanoparticles (NPs) on a scale ranging from 1 to 100 nm. The NPs of metal and metal oxides are usually required in industry. Several types of metal and metal oxide NPs such as aluminium, nickel, silver, copper, copper oxide, iron, iron oxide, cerium dioxide, titanium dioxide and zinc oxide (ZnO) are commonly known [1,2]. The NPs can be prepared by several physical, chemical and biological methods, but physical and chemical methods are associated with high energy demand, and sometimes generating poisoned and perilous chemicals, which may lead to related dangers [3,4]. To minimize these problems, a safe, cost-effective and less hazardous synthesis procedure has already been developed by modern scientists, namely the biological or green method using plant extract with a low concentration of the chemicals. Among all metal oxides, zinc oxide nanoparticles (ZnO NPs) have drawn more attention for their safe and inexpensive production and preparation process [5,6]. ZnO has been enrolled as one of the safest metal oxides by the U.S. Food and Drug Administration [7]. There are a lot of applications of ZnO in engineering, biological and medicinal fields. ZnO NPs have several engineering applications, such as in solar cells [8–10], gas sensors [11], chemical sensors [12], biosensors [13] and photodetectors [14], whereas, in biological and medicinal applications, ZnO NPs have cytotoxic activity [15], antimicrobial and fungicidal activities [16], anti-inflammatory activity, and wound-healing, antidiabetic [17,18] and chemiluminescent properties [19,20].

Studies have supported the synthesis of ZnO NPs in several nanosized from various plant parts like the leaf, flower, seed, fruit, root, rhizome, stem, bark, shell and peel extracts. For example, researchers have used: leaf extracts of *Pandanus odorifer* [21], *Eucalyptus globulus* [22], *Aloe barbadensis* [23], *Sechium edule* [24], *Saponaria officinalis* [25], *Annona squamosal* [26], *Artocarpus heterophyllus* [27], *Mangifera indica* [28] and *Laurus nobilis* [29]; flower extracts of *Trifolium pretense* [30], *Anchusa italic* [31] *and Punica granatum* [32]; seed extracts of *Cuminum cyminum* [33] and *Pongamia pinnata* [34]; fruit extracts of *Emblica Officinalis* [35], *Borassus flabellifer* [36] and *Artocarpus gomezianus* [37]*;* root extracts of *Rubus fairholmianus* [38] and *Withania somnifera* [39]; rhizome extracts of *Zingiber officinale* [40] and *Bergenia ciliate* [41]; stem extracts of *Phyllanthus embilica* [42]; bark extracts of *Cinnamomum verum* [43] *and Albizia lebbeck* [44]; and peel extracts of *Punica granatum* [45], *Musa sapientum* [46] and so on.

Previously, we have reported the green synthesis of Ag NPs for enhanced antibacterial activity using *Cocos nucifera* leaf extract [47]. As a continuation of this work, this present study illustrates the green synthesis of ZnO NPs using *Cocos nucifera* leaf extract with profound antimicrobial, antioxidant and photocatalytic activity. *Cocos nucifera* is a perennial tree that grows in tropical seashore areas; the plant is approximately 30 m high and its leaf is approximately 4 m long [48]. This plant grows best in high rainfall areas and soils with pH 5.5–7 [49]. It has various medicinal uses and properties; for example, antidiarrheal, antirheumatic, aphrodisiac, cytotoxic, diuretic, emetic, emollient, hypotensive, kidney treatment, poultice and vermicide properties [50]. *Cocos nucifera* is a fascinating plant with diverse uses ranging from domestic to therapeutic. In the endosperm (coconut meat), endocarp (coconut hard shell) and leaf extract, the existence of phytochemicals such as tannin, saponin, alkaloid, phenol, flavonoid and volatile oil was determined. Only in the case of the leaf extract of the plant, alkaloid, tannin, saponin and flavonoid were identified [51]. Phytochemicals such as alkaloids act as capping and reducing agents to prepare ZnO NPs [33,52] using Zn-salt, e.g. Zn(NO_3_)_2_.6H_2_O.

Several research works have already studied *Cocos nucifera*. Roopan *et al*. [53] studied phytoconstituents, biotechnological applications and nutritive aspects of coconut (*Cocos nucifera*). Satheshkumar *et al*. [54] used curry leaves extracted with coconut water to synthesize ZnO NPs and observed photocatalytic dye degradation and antibacterial activity. Priyatharesini *et al*. [55] used *Cocos nucifera* male flower extract to synthesize ZnO NPs and analysed its antimicrobial activity. Krupa [56] used the endosperm of *Cocos nucifera* (coconut water) to synthesize ZnO NPs and studied tetraethoxysilane sol–gel coatings for combating microfouling. But, still, there is no report on the green synthesis of ZnO NPs using *Cocos nucifera* leaf extract. In this work, we have developed a simple, cost-effective and green approach for the preparation of ZnO NPs using *Cocos nucifera* leaf extract. The structural, morphological and optical properties of the NPs are well explored. Finally, the antimicrobial, antioxidant and photocatalytic activities of the prepared ZnO NPs are studied, with potent outcomes.

## 2. Material and methods

### 2.1. Chemicals and reagents

Fresh *Cocos nucifera* leaves were collected from the local area of Cumilla, Bangladesh. Reagent-grade (purity ≥ 98%) Zn(NO_3_)_2_.6H_2_O and NaOH pellets were purchased from Fluka Analytical, Sigma-Aldrich, Germany. Mueller–Hinton agar and potato dextrose agar were purchased from HiMedia Laboratories Pvt. Ltd., Mumbai, India. Methylene blue (MB) dye, methanol, 2, 2-diphenyl-1-picrylhydrazyl (DPPH) and ascorbic acid were purchased from Merck, Germany. All reagents and chemicals were used as received, with no further purifications.

### 2.2. Preparation of leaf extract

**Figure 1.**
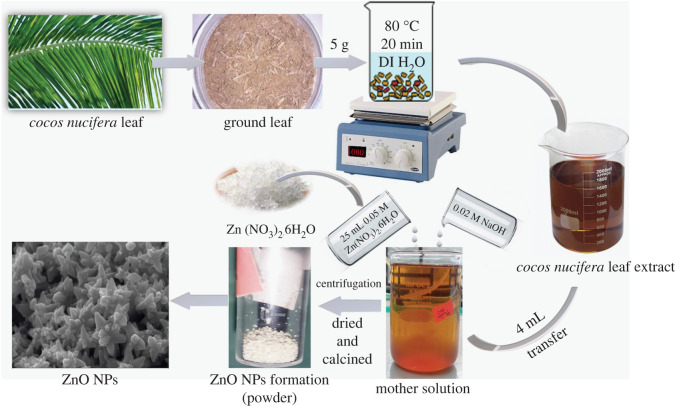
Preparation of ZnO NPs using *Cocos nucifera* leaf extract.

The fresh leaves of *Cocos nucifera* were washed several times by using deionized water to remove dirt particles. After washing, the leaves were left to sun dry and then ground to a fine powder with a mortar. The fine powder leaves (about 5 g) were placed in a 250 ml beaker, mixed with 50 ml of deionized water and heated at 80°C for 20 min. Then, the mixture was filtered into another beaker with Whatman no.1 filter paper and the extract was formed at this stage according to the literature [47,57–59]. The extract was then cooled down and stored in the refrigerator (4°C) for utilization in the synthesis of ZnO NPs, as demonstrated in figure 1.

### 2.3. Preparation of zinc oxide nanoparticles

For the preparation of ZnO NPs, 25 ml of 0.05 M aqueous solution of Zn(NO_3_)_2_.6H_2_O was mixed with 4 ml of the prepared aqueous leaf extract of *Cocos nucifera* in a 250 ml beaker, as demonstrated in figure 1 [47,57–59]. Then, the pH of the mixture was adjusted to 12 by the drop-wise addition of 0.02 M aqueous NaOH solution. The total solution, known as the mother solution, was stirred for 3 h with a magnetic stirrer at ambient temperature and then centrifuged by a high-speed Benchtop centrifuge machine (model no. H3–18 K, Kecheng, China) at 8000 rpm for 20 min. This resulted in a solid product with a light-orange colour, which was then dried overnight in an oven (model no. LDO-060E, Labtech, Korea) at 60°C. After that, the solid product was collected. Finally, the light-orange product turned into a white powder when it was calcined in a muffle furnace (model no. FTMF-703, SCI FINETECH, Korea) at 550°C for 30 min. The white powder was collected in a small sample vial after cooling and stored in a desiccator for further use.

### 2.4. Characterization techniques for the prepared zinc oxide nanoparticles

To characterize the prepared ZnO NPs, we used diverse analytical tools such as ultraviolet–visible **(**UV–Vis**)** spectroscopy, X-ray diffraction (XRD) analysis, Fourier transform infrared (FTIR) spectroscopy, energy-dispersive X-ray (EDX) spectroscopy and scanning electron microscopy (SEM). A UV–Vis spectrophotometer (UV prove 1800, Shimadzu, Japan) was used primarily to confirm the formation of ZnO NPs, and the spectrum was recorded in the range of 300–500 nm by using deionized water as a reference. XRD was used to identify the phase and information of unit cell dimensions of the prepared materials [60]. XRD of the powdered ZnO NPs was conducted by Explorer GNR, using monochromatic Cu Ka radiation (1.5419 Å) operated at a voltage of 40 kV and current of 30 mA, with 2*θ* angle (30°–80°) pattern and scan speed of 2°/min. All likely diffractions were determined by scanning the sample from 2*θ* angles to account for all possible orientations of the powder sample. These diffraction peaks were converted to d-spacings, which allow the specific identification of each material [61]. Estimation of crystalline size (D) of the prepared ZnO NPs was calculated by Debye–Scherrer formula [62] shown below:

2.1 $$D=\frac{k\lambda }{\beta \cos\theta },$$

where *k* (value 0.9) is the shape factor (dimensionless), *λ* is the X-ray wavelength of 1.5419 Å, *β* is the full width at half maximum (FWHM) in radian and *θ* is the Bragg angle in radian.

The FTIR spectrophotometer (IRAffinity-1S, Shimadzu, Japan) was employed to identify the characteristics of functional groups coming from the conjugation between nanomaterial and the adsorbed biomolecules [63]. The FTIR spectrum of the prepared powdered sample was recorded in a wide range of wavenumbers (400–4000 cm^−1^) with 20 scans and 2 cm^−1^ resolution, which used the Happ-Genzel apodization function and KBr pellet method. The surface morphology and elemental composition of the prepared material were studied by SEM equipped with EDX (Model: EVO18, Carl Zeiss Microscopy, USA). A high-quality surface image of the sample was obtained by scanning it with a focused beam of electrons from an electron gun applying an acceleration voltage of 15 kV.

### 2.5. Antimicrobial screening of the prepared zinc oxide nanoparticles

Antimicrobial screening is an important method of analysis of the inhibitory effects of compounds against microorganisms [64]. There are a few laboratory methods available to evaluate the antimicrobial activity of a compound. The agar dilution or disc diffusion method is the most common [65]. Antimicrobial screening of ZnO NPs (50 µl dose and concentration 150 µg disc^−1^) was assessed against various bacterial and fungal strains. Three gram-positive bacterial strains such as *Staphylococcus aureus* (cars-2), *Bacillus megaterium* (*BTCC-18*) and *Bacillus cereus* (carsgp-1), as well as two fungal strains, namely *Aspergillus niger* (carsm-3) and *Trichoderma harzianum* (carsm-2), were used in the agar well diffusion method, similar to our previous report [66]. Mueller–Hinton agar (HiMedia, India) was used to form an agar medium to culture bacteria and potato dextrose agar medium (HiMedia, India) was used to culture fungal strains. As standards, *Ceftriaxone* (10 µl) was used for bacterial strains and *amphotericin-B* (10 µl dose and 50 µg disc^−1^) was used for fungal strains [66]. After placing the sample in a culture medium, the discs were incubated for 24 h at 37°C for bacteria and 48 h at 26°C for fungi. The antimicrobial activity was determined by measuring the zone of inhibition (ZOI).

### 2.6. Photocatalytic behaviour of zinc oxide nanoparticles

The ZnO NPs can act as photocatalysts because they exhibit photocatalytic activity under irradiation of sunlight [5,24]. For this experiment, we used a 50 mg L^−1^ aqueous solution of MB dye with a 5 mg/20 ml catalytic load of ZnO NPs. After mixing both solutions (dye and catalyst), the blue dye solution turned into a colourless solution within 1 h. The UV absorption was taken after 0, 10, 15, 30 and 60 min. The percentage of degradation was calculated by the following equation:

2.2 $$\text{\%}\;\mathrm{of}\;\mathrm{degradation}=\left(\frac{C_{i}-C_{f}}{C_{i}}\right)\times 100,$$

where C*_i_* and C*_f_* are the initial and final concentrations of dye that degrade with time.

### 2.7. DPPH radical scavenging activity assay

A DPPH radical scavenging assay was performed to determine the ability of the prepared ZnO NPs to scavenge free radicals. The ability of NPs to inhibit oxidation was tested by decolourizing a methanol solution of DPPH. In methanol solution, DPPH creates a violet/purple colour, which fades to shades of yellow in the presence of antioxidants. A 0.1 mM DPPH in methanol solution was prepared and 2.4 ml of it was combined with 1.6 ml of extract in methanol at varying concentrations (6.25–1200 µg ml^−1^). The reaction mixture was vortexed completely and kept at room temperature for 30 min in the dark. At 517 nm, the absorbance of the mixture was determined spectrophotometrically. Ascorbic acid was used as a standard. The following equation was used to compute the percentage of DPPH radical scavenging activity:

2.3 $$\text{\%}\mathrm{DPPH}\;\mathrm{radical}\;\mathrm{scavenging}\;\mathrm{activity}=\left[\frac{\left(A_{0}-A_{1}\right)}{A_{0}}\right]\times 100,$$

where the absorbance of the control is *A*_0_ and the absorbance of the sample is *A*_1_.

The per cent of inhibition was then plotted against concentration, and the IC_50_ was derived from the graph. At each concentration, the experiment was performed three times [67].

## 3. Results and discussions

### 3.1. Mechanism of zinc oxide nanoparticles formation

**Figure 2.**
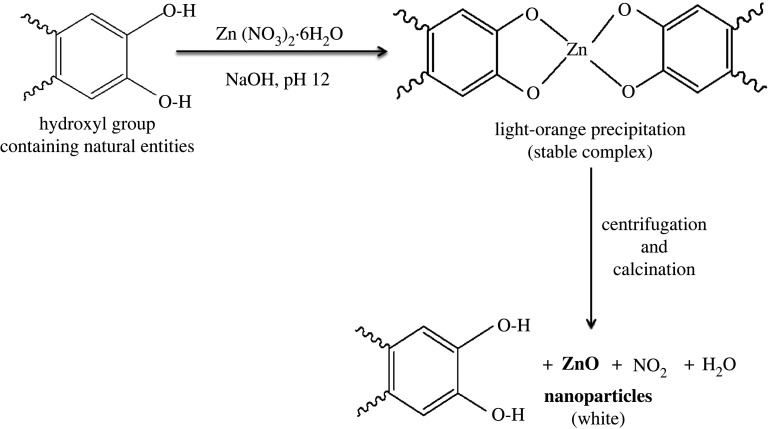
Proposed mechanism of ZnO NPs formation.

There are several proposed mechanisms for the formation of ZnO NPs in the green synthesis approach [21–46,68,69]. In the present work, we used the aqueous extract of *Cocos nucifera* leaf as both the stabilizing and natural reducing agent for ZnO NPs preparation. The phytochemical screening of this leaf demonstrated the existence of various phytochemicals such as alkaloids, resins, steroids, glycosides, terpenoids, flavonoids, polyphenols and aromatic hydrocarbons [33,52]. The presence of these phytochemicals in *Cocos nucifera* leaf plays an important role in the NPs preparation, acting as reducing and capping agents. Figure 2 shows the probable reaction mechanism for the formation of ZnO NPs in which aromatic hydroxyl groups present in the phytochemicals and polyphenols are attached to the Zn^2+^ ions from Zn(NO_3_)_2_.6H_2_O to form a stable complex system. This complex system releases ZnO NPs after centrifugation and calcination [70–72].

### 3.2. Ultraviolet–visible spectroscopy analysis

**Figure 3.**
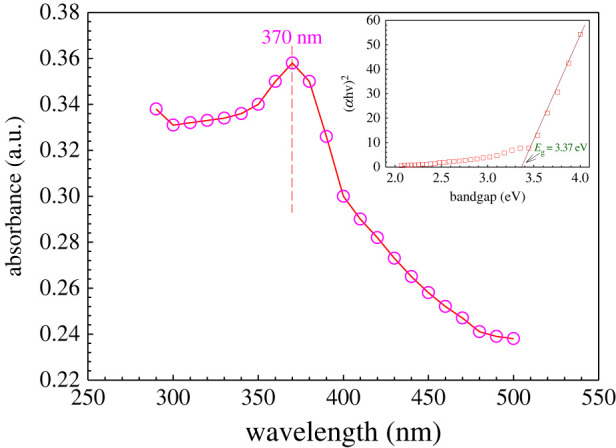
UV–Vis spectrum of ZnO NPs prepared using *Cocos nucifera* leaf extract.

ZnO NPs generally show UV absorption bands in the *λ*_max_ ranges from 355 to 380 nm [73,74]. Figure 3 shows the absorption intensity of the prepared ZnO NPs measured in the wavelength range from 300 to 500 nm. The ZnO NPs prepared using *Cocos nucifera* leaf extract showed *λ*_max_ at 370 nm, which is supported by the literature [65–67]. The bandgap energy of ZnO NPs was found to be 3.37 eV as calculated by using Tauc's plot, which is similar to the reported bandgap energy of ZnO (wide band gap 3.10–3.39 eV) [31,33,75,76]. These findings primarily confirm the formation of ZnO NPs following our approaches.

### 3.3. X-ray diffraction analysis

**Figure 4.**
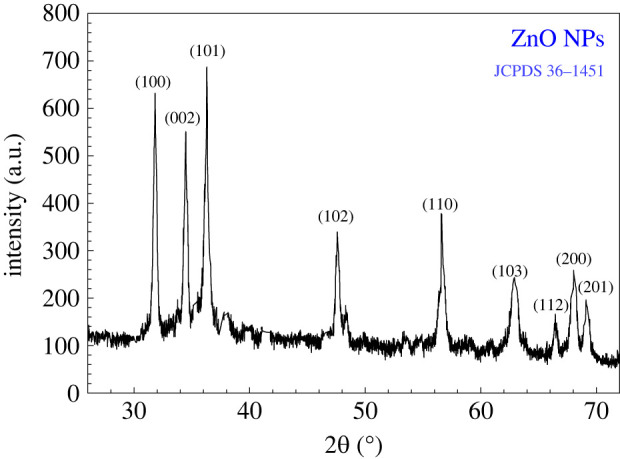
XRD pattern of ZnO NPs prepared using *Cocos nucifera* leaf extract.

The white powder obtained from the preparation step of the material was subjected to XRD analysis and the corresponding XRD patterns are shown in figure 4. The XRD patterns revealed nine diffraction peaks appearing at 2*θ* angles of 31.83°, 34.46°, 36.28°, 47.58°, 56.62°, 62.91°, 66.46°, 68.06° and 69.10° corresponding to the Miller indices of 100, 002, 101, 102, 110, 103, 112, 200 and 201, respectively. According to JCPDS card no: 36–1451, the obtained patterns identified our prepared material as ZnO with a hexagonal wurtzite structure, space group: P63mc, unit cell volume: 47.62, unit cell parameters: *a* = *b* = 3.25 Å and *c* = 5.21 Å, and *α* = *β* = 90° and *γ* = 120°. The obtained XRD patterns are quite comparable with previous reports [31,74]. The average crystal size of the prepared ZnO NPs was calculated by using the Debye–Scherrer equation (§2.4, equation (2.1)) and was found to be 16.6 nm (range: 11.9–24.1 nm).

### 3.4. Fourier transform infrared spectroscopy analysis

**Figure 5.**
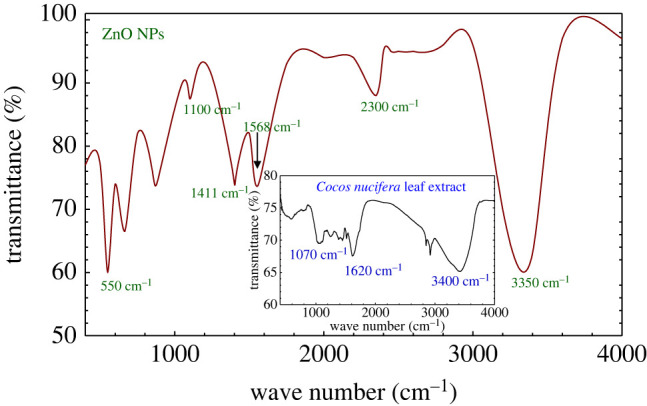
FTIR spectrum of ZnO NPs prepared using *Cocos nucifera* leaf extract. The inset shows the FTIR spectrum of *Cocos nucifera* leaf extract.

The FTIR spectrum of the prepared ZnO NPs using *Cocos nucifera* leaf extract is illustrated in figure 5. The inset of figure 5 shows the FTIR spectrum of *Cocos nucifera* leaf extract. This spectroscopic measurement was carried out to identify the functional groups of the possible biomolecules responsible for the capping and efficient stabilization of the ZnO NPs. According to the literature [77], the peaks that appeared at 3200–3600 cm^−1^ in the FTIR spectrum can be corroborated by the O−H stretching alcohols, stretching vibrations of the primary and secondary amines, and C−H stretching of alkanes. The peaks observed at 1568, 1411 and 1100 cm^−1^ were due to the C = C stretching in the aromatic ring in polyphenols and aliphatic amines, while the peak at 2300 cm^−1^ originated from di-substituted alkynes, and the peak at 550 cm^−1^ was from the hexagonal phase of ZnO [77,78]. As mentioned previously, the *Cocos nucifera* leaf contains alkaloids, steroids, terpenoids, flavonoids, polyphenols and aromatic hydrocarbons. As a consequence, the results of the FTIR analysis indicate that the functional groups present in the biomolecules of leaf extract, as well as phytocompounds such as alkaloids, steroids, terpenoids, flavones, polyphenols and aromatic hydrocarbons, may also act as reducing and capping agents for ZnO NPs formation and prevent agglomeration of the NPs in the aqueous extract medium [47,79].

### 3.5. Energy-dispersive X-ray analysis

**Figure 6.**
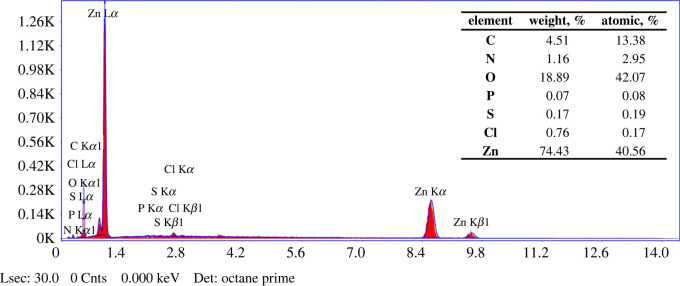
EDX spectrum and elemental composition of ZnO NPs prepared using *Cocos nucifera* leaf extract.

The elemental composition of ZnO NPs was obtained from EDX analysis. Figure 6 shows the existence of chemical elements and their composition in the prepared ZnO NPs. The presence of a large percentage of Zn and O is indicative of ZnO formation. As expected, the atomic percentages of Zn and O are almost equal (ZnO in 1 : 1 ratio) and the highest percentage of C, along with some other elements such as N, P, S and Cl, originated from the biomolecules of *Cocos nucifera* leaf. The presence of Zn at a high percentage, whereas C and other elements are at lower percentages, indicates that plant phytochemical groups were involved in reducing and capping the ZnO NPs.

### 3.6. Scanning electron microscopy analysis

**Figure 7.**
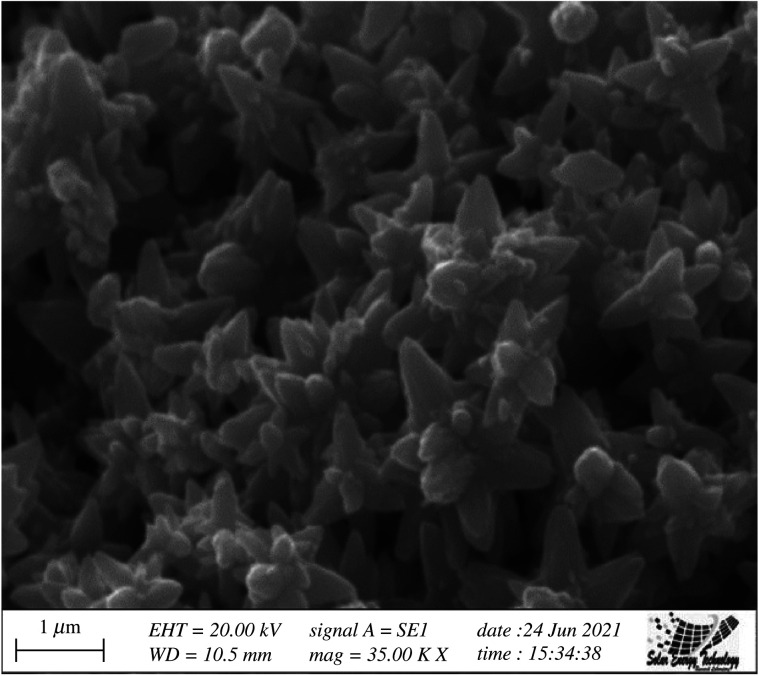
SEM image of ZnO NPs prepared using *Cocos nucifera* leaf extract.

The surface morphology of the prepared ZnO NPs was studied by SEM analysis. The SEM image of ZnO NPs is depicted in figure 7, which shows a uniform distribution of flower-shaped ZnO molecules. The particle size of ZnO NPs of about 15 nm was calculated from the SEM image using ImageJ software, which is in agreement with the calculated particle size (16.6 nm) from XRD data. The hydrogen bonding and electrostatic interaction between bioorganic capping molecules and NPs have resulted in them accumulating together [80]. Moreover, the SEM image showing ZnO NPs revealed that they are not in direct contact with each other, which signifies the stabilization of NPs by capping agents [81].

### 3.7. Antimicrobial screening analysis

#### 3.7.1. Mode of action

**Figure 8.**
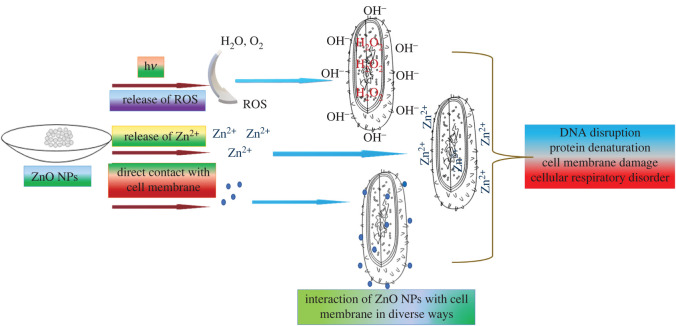
Mode of action of ZnO NPs against microbes.

The ZnO NPs showed good antimicrobial activity against a wide variety of microbes, including bacteria and fungi. The prepared NPs interact with the cell membrane of microbes in different pathways, such as through the release of reactive oxygen species (ROS), the release of Zn^2+^ and direct contact with the cell membrane. This process damages the cell through DNA disruption, protein denaturation, cellular respiratory disorder, cell membrane damage and so on. Figure 8 illustrates the discussed mechanism, which is also proposed in the literature [82,83].

#### 3.7.2. Antimicrobial activity of zinc oxide nanoparticles

Antimicrobial activity was studied against three bacterial and two fungal pathogenic strains, as shown in table 1. The highest ZOI of ZnO NPs was found to be 14 mm for fungal pathogenic strains of *T. harzianum*, which causes infection in human recipients of renal transplant [84]. Synthesized ZnO NPs show moderate antimicrobial activity (greater than or equal to 10 mm) with gram-positive bacteria *S. aureus* (10 mm) and fungus *A. niger* (10 mm), and slight antimicrobial activity (8–9 mm) against *B. megaterium* and *S. aureus*.

**Table 1.** ZOI diameters (mm) of ZnO NPs, ceftriaxone and amphotericin-B against tested bacterial and fungal strains.

material	gram-positive bacteria			fungi	
	*B. megaterium*	*S. aureus*	*B. cereus*	*A. niger*	*T. harzianum*
ZnO NPs	08	10	08	10	14
ceftriaxone	50	40	20	–	–
amphotericin-B	–	–	–	08	17

#### 3.7.3. Photocatalytic behaviour of zinc oxide nanoparticles

**Figure 9.**
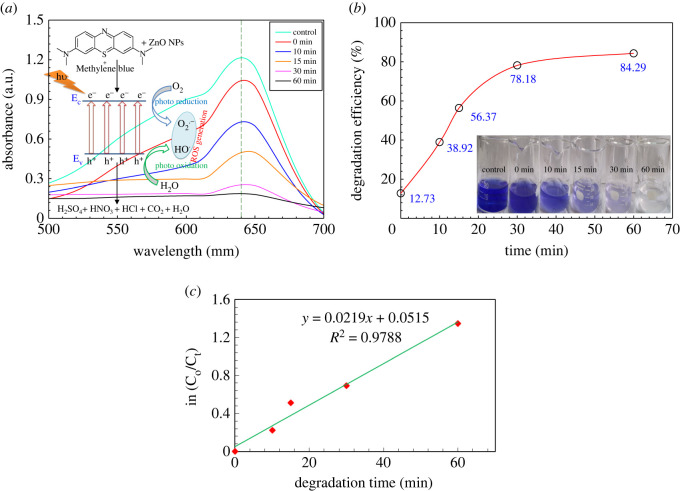
(*a*) UV–Vis spectra showing the degradation of MB dye through the photocatalytic activity of the prepared ZnO NPs; (*b*) percentage of degradation of MB dye with respect to time, where the inset figure illustrates the colour change with time during the degradation of MB dye with ZnO NPs; (*c*) shows the reaction kinetics.

Figure 9*a* demonstrates the photocatalytic behaviour of the prepared ZnO NPs, which represents the degradation of dye concentration at different time intervals. Figure 9*b* represents the percentage of dye degradation with respect to time, with a clear view (inset) of the colour change during the reaction. The initial absorbance of the dye was compared with the final absorption after mixing the prepared ZnO NPs with the dye. It was shown that the absorbance degrades graphically at 640 nm. The mixed solution was discoloured after 1 h and MB dye degraded a maximum of 84.29% by the ZnO NPs. A possible mechanism has been proposed [85,86] for this degradation process, namely the production of ROS by photo-oxidation (hydroxyl radical generation) and photo-reduction (peroxide radical generation) processes [87]. The reaction rate can be calculated using the first-order kinetic equation ln(*C*_0_/*C_t_*) = *kt*, where *C*_0_ and *C_t_* are the initial and final concentrations of ZnO NPs, *k* is the rate constant, which is equal to 0.0219 min^−1^, and *t* is the degradation time showed in figure 9*c*. Finally, this ROS degraded the dye into mineral acids, CO_2_ and water. The probable mechanism is embedded in the inset of figure 9*a*.

#### 3.7.4. Antioxidant activity assay

**Figure 10.**
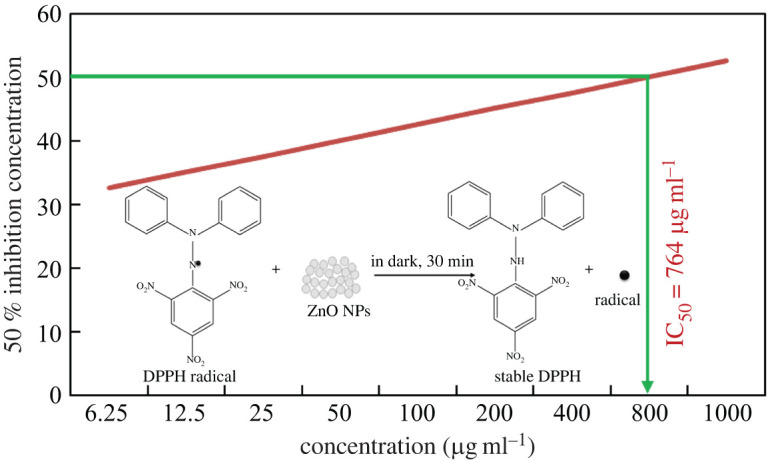
DPPH free radical scavenging activity of the prepared ZnO NPs, showing the IC_50_ value. Inset shows the mechanism for DPPH free radical scavenging activity of ZnO NPs.

Figure 10 demonstrates the DPPH free radical scavenging activity of the prepared ZnO NPs, showing the 50% inhibition (IC_50_) value. The inset of the same figure illustrates the mechanism for DPPH free radical scavenging activity of ZnO NPs following that of Murali *et al*. [88]. At 517 nm, the absorbance of DPPH decreased with the increase in concentration of ZnO NPs. This result indicates that the prepared ZnO NPs can inhibit oxidation due to the transfer of electron density located at the oxygen atom to the nitrogen atom in the DPPH free radical, which contains an odd electron by n→π* transition [89,90]. The required concentration of the prepared ZnO NPs to show IC_50_ of DPPH was found to be 764 µg ml^−1^.

## 4. Conclusion

In this study, we have successfully prepared ZnO NPs with an average size of 16.6 nm, using *Cocos nucifera* leaf extract by a simple, inexpensive and green approach. The prepared NPs were identified and characterized by different techniques, such as UV–Vis spectroscopy, XRD, FTIR, EDX and SEM analyses. The aqueous solution of the prepared ZnO NPs showed absorption maxima, *λ*_max_, at 370 nm in UV–Vis spectroscopic measurement. XRD analysis identified the formed ZnO NPs with a hexagonal wurtzite structure. FTIR analysis indicated the presence of reducing biomolecules associated with organic functional groups responsible for the encapsulation and stabilization of the NPs. The elemental composition obtained from EDX analysis supports the formation of the desired ZnO NPs. The antimicrobial study of the prepared ZnO NPs showed that the material is very active against various pathogenic bacteria and fungi. The prepared NPs showed high photocatalytic activity and moderate antioxidant activity. Thus, we can conclude that the prepared ZnO NPs could be used in biomedical, medicinal and pharmaceutical applications, and also as photocatalysts in the dye degradation process.

## Data Availability

Our data are deposited at Dryad Digital Repository: https://doi.org/10.5061/dryad.tht76hf27 [91].
